# Relationship between antibiotic resistance genes and metals in residential soil samples from Western Australia

**DOI:** 10.1007/s11356-016-7997-y

**Published:** 2016-11-07

**Authors:** Charles W Knapp, Anna C Callan, Beatrice Aitken, Rylan Shearn, Annette Koenders, Andrea Hinwood

**Affiliations:** 1 Department of Civil & Environmental Engineering, University of Strathclyde, Glasgow, Scotland G1 1XJ UK; 2 School of Medical and Health Sciences, Edith Cowan University, Joondalup, WA 6027 Australia; 3 Centre for Ecosystem Management, Edith Cowan University, Joondalup, WA 6027 Australia

**Keywords:** Antimicrobial resistance, Antibiotic resistance, Garden soil, Toxic heavy metal, qPCR

## Abstract

Increasing drug-resistant infections have drawn research interest towards examining environmental bacteria and the discovery that many factors, including elevated metal conditions, contribute to proliferation of antibiotic resistance (AR). This study examined 90 garden soils from Western Australia to evaluate predictions of antibiotic resistance genes from total metal conditions by comparing the concentrations of 12 metals and 13 genes related to tetracycline, beta-lactam and sulphonamide resistance. Relationships existed between metals and genes, but trends varied. All metals, except Se and Co, were related to at least one AR gene in terms of absolute gene numbers, but only Al, Mn and Pb were associated with a higher percentage of soil bacteria exhibiting resistance, which is a possible indicator of population selection. Correlations improved when multiple factors were considered simultaneously in a multiple linear regression model, suggesting the possibility of additive effects occurring. Soil-metal concentrations must be considered when determining risks of AR in the environment and the proliferation of resistance.

## Introduction

Antibiotic resistance (AR) has become one of the most significant problems to threaten populations globally (WHO 2014), as the prevalence of multidrug-resistant bacterial infections continues to increase (Canton 2009; Tenover 2006). Generations of newly developed antibiotics lose their efficacy against many bacterial infections within a few years after their introduction (Davies and Davies 2010), and along with the difficulty to develop and supply new effective antibiotics (Shlaes 2010), antibiotic resistance threatens the effectiveness of chemical therapies. The rapid appearance of resistance traits suggests that a reservoir of resistance traits exists, and factors, other than those in the clinical setting, influence the dissemination of these genes. As such, attention has shifted towards the environment (Martinez 2008).

Understanding the link between AR and environmental conditions remains crucial. The assumptions that AR solely originates from the misuse or overuse of antibiotics are no longer acceptable; even after efforts have been made for reduced and more controlled antibiotic applications, AR continues to increase. Resistance occurs not only by selective pressure caused by antibiotics but also due to contaminants that promote the dissemination of genetic elements by cross-resistance and co-resistance processes (Ashbolt et al. 2013; Baker-Austin et al. 2006; Berg et al. 2010; Perry and Wright 2013). Bacterial communities respond genetically to pollutants via evolved mechanisms for their self-protection (Alonso et al. 2001); in particular, potentially toxic metals contribute to the stress response (Ashbolt et al. 2013; Beaber et al. 2004; Berendock et al. 2015). As such, the environment acts both as a reservoir of resistance traits and a bioreactor containing chemical stressors and opportunities for genetic exchange. The potential for these traits to disseminate to clinically relevant pathogens becomes a consequence.

The relationship between metal tolerance (or resistance) and AR has been known; reviews can be found in the literature (e.g., Baker-Austin et al. 2006; Martinez 2009; Seiler and Berendonk 2012; Perry and Wright 2013). Certain metals, albeit toxic at high concentrations, contribute to the biochemical health of microorganisms; mechanisms exist for their regulation and handling. However, clinically relevant infections have been found resistant to multiple antimicrobials, including metals, by susceptibility assays (e.g., Bass et al. 1999; Dhakephalkar and Chopade 1994; Ghosh et al. 2000; Guo et al. 2014; Marques et al. 1979), suggesting that a link between genetic traits exists.

In the environment, relationships between metals and AR proliferation were first noticed in highly contaminated areas, including outflows of insufficiently treated wastewater and biosolids (Graham et al. 2011; Knapp et al. 2012; Su et al. 2015), land application of agricultural wastes (Ji et al. 2012; Li et al. 2015; Zhu et al. 2013), industrial contamination (Abella et al. 2015; Graham et al. 2011; Hu et al. 2016; Knapp et al. 2012; Stepanauskas et al. 2005; Wright et al. 2006) and direct application via metal exposure experiments (Berg et al. 2005, 2010; Knapp et al. 2011; Stepanauskas et al. 2006). AR presence in these cases has often been indicative of exposure to elevated metal pollution; however, limited information exists on less-impacted soils and requires further investigation to ascertain basal risks of AR, especially in residential areas.

Here, we compared total metal conditions and the presence of AR genes from soil samples collected from residences in a range of urban and regional areas in Western Australia. This investigation not only determines the risk of AR bacteria in residential garden soils but also elucidates the feasibility of predicting AR in soils, especially where potentially toxic metals exist at lower concentrations.

## Methods

### Sample collection, preparation and characterisation

Soil samples were collected from residential areas in Western Australia as part of the Australian Maternal Exposure to Persistent Toxic Substances (AMETS) study (Callan et al. 2013). The aim of the original study was to assess whether residential soil and dust were sources of pollutant exposure to pregnant women; sampling procedures were designed to optimise and to minimise sample handling and potential interference by vegetation. Participants resided in either the Perth metropolitan area (10% samples) or regional towns in Western Australia (remaining 90%) including Albany, Bunbury, Bridgetown, Nannup, Busselton, Dunsborough, Margaret River, Collie, Esperance, Geraldton, Kalgoorlie and Port Hedland, which are categorised as Inner Regional Australia, Outer Regional Australia or Remote by the Australian Standard Geographical Classification Remoteness Area system (Australian Institute of Health and Welfare 2004). Eighty samples were taken from areas with Mediterranean climate, and ten considered subtropical or tropical climate.

Soils were collected by volunteering homeowner sampling at least four bare areas of soil. They were instructed to take surface soil samples using a trowel or spoon in the boundary of the residential property and consolidated into a labelled plastic bad provided to them. No vegetation was required to be removed from the samples. There were no known contaminants on these properties; industries in the area included mining and agriculture.

Soil samples were collected between 2008 and 2011. They were mixed thoroughly, air dried (2 days at room temperature) and sieved (1.0-mm mesh). To minimise cross-contamination, the sieves were cleaned with a brush and fresh paper tissue and rinsed with acetone between samples. The samples were stored in sealed containers at room temperature.

Soil characterisation was undertaken on a subset of soil samples (*n* = 59, based on the mass of sample available). Soil particle size analysis was undertaken to determine the particle distribution using the Bouyoucos Hydrometer method based on established protocols (Sheldrick and Wang 1993; Day 1965). The organic matter content in soil samples was determined using weight loss on ignition (Schulte and Hopkins 1996; Schollenberger 1945). The pH and electrical conductivity of the soil samples in solution was measured using precalibrated pH and conductivity meters (Van Reeuwijk 2002; Rhoades 1982).

### Chemical analyses

Approximately 1 g of soils was acid-digested for metal content with 1:1:1 HNO_3_, 30% H_2_O_2_ and concentrated HCl at 95 °C (USEPA 2002). Once cooled, digested samples were filtered (Whatman No. 41 filter paper) and brought to 100 mL final volume with deionised water.

Samples were analysed using a Varian Vista Pro Inductively Coupled Plasma Optical Emission Spectrometry (ICP-OES) (Varian Analytical Instruments, Australia) at the ECU laboratories. The metals analysed included aluminium, arsenic, cobalt, copper, lead, manganese, mercury, nickel, selenium, uranium, vanadium and zinc. The instrument was calibrated using five standard solutions achieving a standard calibration of *r* ^2^ > 0.99; standards and calibrations were checked several times during the analysis procedure using certified reference material RM 8704 (river sediment with 5 mg/L elemental spike; representing the concentration ranged needed for this study). Recoveries for elemental spikes ranged between 85 and 100% for all analyses, and replicate results remained within 10% coefficient of variation.

Ideally, knowing the extractable element content would have been useful for this analysis. However, the nature of prolonged sample storage in the archive and the natural soil ‘ageing’ process would have prevented accurate determinations. Given that, it was decided that the analysis would not have been worth the depletion of soils since only limited amounts remained in storage.

### Molecular microbial analysis

Molecular biological analysis of each sample involved DNA extraction and purification using the PowerLyzer PowerSoil DNA Isolation Kit MB12855-50 (GeneWorks Pty Ltd., Hindmarsh, South Australia) with a Powerlyzer 24 bench-top, bead-based homogeniser. Several trials were undertaken to find a bead beating rate that provided an optimum yield with minimal DNA shearing. For this particular study, homogenisation at 3000 rpm for 45 s, followed by an incubation at 70 °C for 10 min, then another bead beating step of 3000 rpm for 25 s gave optimum yield with minimal DNA shearing for most samples. All DNA extraction protocol steps were performed at room temperature, aside from bead beating steps, which were performed in a cold room (4 °C). The degree of DNA shearing was examined using gel electrophoresis, and DNA extraction quality was verified using a NanoDrop spectrophotometer.

In each sample, quantitative PCR (Bio-Rad iCycler, Hampstead, UK) enumerated genes related to beta-lactam (*bla*_TEM_, *bla*_CTX_, *bla*_SHV_ and *bla*_OXA_ (Knapp et al. 2010)), sulphonamide (*sul*1, *sul*2 and *sul*3 (Pei et al. 2006)) and tetracycline resistance (*tet*M and *tet*W (Peak et al. 2007; Smith et al. 2004)) genes. Additionally, to screen as many tetracycline genes as possible, multiplex qPCR assays were used (Ng et al. 2001), where tet1 (*tet*B, *tet*C and *tet*D) and tet2 (*tet*A, *tet*E, *tet*G) surveyed many efflux genes, tet3 (*tet*K, *tet*L, *tet*M, *tet*O and *tet*S) represented a mix of efflux and ribosomal protection genes, and tet4 included *tet*A(P) (efflux pump), *tet*X (enzyme) and *tet*Q (ribosomal protection protein). The selection of genes was not intended to be exhaustive, rather they provided key ‘biomarkers’ that have been routinely measured previously (e.g., Graham et al. 2011; Knapp et al. 2010; Knapp et al. 2011; Pei et al. 2006; Pruden et al. 2006). 16S ribosomal ribonucleic acid (16S rRNA) genes were also measured to determine relative abundances of ARG as a surrogate measure of ‘total bacteria’, using the 338F-805R primer pair (5′-3′; with 515F probe) that should minimise non-target binding to plant, animal and fungal DNA (Dorn-In et al. 2015).

Each 20 μL reaction comprised of 10 μL iQ Supermix (Bio-Rad), 7 μL molecular-grade water (Qiagen; Hilden, Germany), 1 μL primers (500 nM; Sigma-Aldrich; Haverhill, England), 2 μL of DNA template and SYBR Green I for fluorescence detection. Temperature cycles involved 10 min at 95 °C for initial denaturation and 40 cycles of denaturation (1 min, 95 °C), primer annealing (30 s, primer-specific temperature (see references listed above)) and elongation and fluorescence detection (30 s, 72 °C). Gene-containing plasmids, each diluted in yeast tRNA solution to 10^1^ to 10^7^ copies per microlitre, were used as (neat) standard controls as prepared by Smith et al. (2004). Gene determinants *tet*A, *tet*B, *tet*O and *tet*Q were used as controls for the *tet* multiplex assays. Before analysis, aliquots of randomly selected samples were serially diluted and analysed by PCR (targeting 16S rRNA gene); the resulting trend lines were compared with those of ‘neat’ standards; the lowest dilution, at which trend lines had comparable slopes with standards and generated minimal within-sample variability, was selected. As such, all samples were diluted 1:100 with molecular-grade water to minimise inhibitory effects on the PCR polymerase enzyme. Post-analytical quality control included a melt curve of PCR products to verify reaction quality (50–95 °C, Δ*T* = 0.1 °C/s).

### Data analysis

ARG data were analysed in two ways: raw abundances (per gram soil extracted) or relative abundances (normalised per 16S rRNA genes). Non-normalised data do not take into account the bacterial population size, meaning a sample containing a high number of ARG can either be attributed to many bacteria present in the samples or a high number of genes existing in the community. This is often used to compare gene flux in run-off and surfacewater scenarios (Knapp et al. 2012). Normalising gene counts to 16S rRNA genes (a surrogate measure of total bacteria) presents an approximate proportion of bacteria that carry the gene of interest.

All statistics involved SPSS™ version 20. Metal concentrations below the limit of detection (LOD) were assigned a value of half the LOD for analysis. Analyses included log transformation of gene values and soil metal concentrations to ensure distribution normality (Kolmogorov-Smirnov test). We predetermined significance level (*α* value) at 10% to account for the variable nature of environmental samples. Bivariate Pearson’s correlations were undertaken to determine the relationships between log-transformed soil metal concentrations and gene values. Multiple linear regression models were generated using log-transformed normalised gene content as the dependent variables and metal concentrations as the independent variables; the metals entered into the model were selected on the basis of bivariate correlations.

## Results and discussion

### Environmental conditions

Soils were collected from 90 sites in Western Australia; the composition of the soils was predominantly sand (≥70%), with pH ranging from slightly acidic to neutral and a range of organic matter content (Table 1). Further, soils were analysed for aluminium, arsenic, cobalt, copper, mercury, manganese, nickel, lead, selenium, uranium, vanadium and zinc content (Callan et al. 2013; Hinwood et al. 2013) (Table 1). While most values ranged within values typical of ‘background’ in Europe (McLaughlin et al. 2010) and Australian soils (NEPC 1999), there were a few minor exceptions. High vanadium (>50 mg/kg), selenium (>200 mg/kg) and mercury levels (>1 mg/kg) were found at a few sites, but they were within Soil Quality Standards for Europe and the National Environment Protection Measures (NEPM) guidelines for ‘no action’ in residential areas in Australia (NEPC 1999). As would be anticipated, the proportion of sand in the soil samples was negatively correlated with the proportion of silt and clay (*r* = −0.857, *p* < 0.001 and *r* = -0.756, *p* < 0.001, respectively) and also with the electrical conductively of the soils (*r* = −0.396, *p* = 0.002) and the presence of organic matter (*r* = −0.439, *p* = 0.001).

**Table 1:** Soil character and metal concentrations (mg/kg) in samples collected throughout Western Australia (*n* = 90)

	Mean (95% CI)	Min–max	<%LOD^a^
% Sand	88.2 (1.9)	70.0–97.5	
% Silt	6.0 (1.3)	0.5–23.0	
% Clay	5.9 (1.0)	0.0–19.5	
pH	7.0 (0.1)	5.7–8.1	
Electrical conductivity (μS/cm)	419 (80)	22–1890	
% Organic matter	6.2 (0.8)	1.5–13.9	
Aluminium	1800 (450)	<2.50–13,000	2.2
Arsenic	4.91 (0.96)	<3.50–22.9	57.8
Cobalt	0.75 (0.11)	<1.00–2.78	76.7
Copper	7.46 (1.83)	<0.04–48.3	13.3
Mercury	1.31 (0.55)	<1.0–17.0	81.1
Manganese	49.9 (13.4)	<0.20–443	2.2
Nickel	2.26 (0.60)	<2.00–19.1	70.0
Lead	9.27 (2.96)	<3.00–96.4	50.0
Selenium	14.6 (8.8)	<3.00–293	71.1
Uranium	21.5 (13.2)	<1.00–592	23.3
Vanadium	13.3 (4.4)	<0.30–97.1	5.6
Zinc	33.2 (8.5)	<0.60–197	7.8

^a^<%LOD indicates proportion of samples below respective analytical limit of detection for that metal

For the metals manganese, nickel, selenium and vanadium, the concentrations of these metals in soil samples were found to be negatively correlated with the proportion of sand and positively correlated with the percentage of silt and clay: Mn (sand, *r* = −0.745, *p* < 0.001; silt, *r* = 0.704, *p* < 0.001; clay, *r* = 0.481, *p* < 0.001), Ni (sand, *r* = −0.480, *p* < 0.001; silt, *r* = 0.466, *p* < 0.001; clay, *r* = 0.293, *p* = 0.023), Se (sand, *r* = −0.339, *p* = 0.009; silt, *r* = 0.362, *p* = 0.005; clay, *r* = 0.165, *p* = 0.211) and V (sand, *r* = −0.755, *p* < 0.001; silt, *r* = 0.723, *p* < 0.001; clay, *r* = 0.474, *p* < 0.001). The concentration of aluminium in the soil samples was negatively correlated with soil pH (*r* = −0.505, *p* < 0.001), with higher concentrations identified in more acidic soils, which may reflect increased solubility.

DNA was extracted from a subset of the soils and analysed for 16S rRNA and selected AR genes (Table 2) and resulted in an array of total gene values ranging from 10^−6^ to 10^−2^ genes/16S rRNA, representing approximately 0.0001 to 1% of the total bacteria. AR gene concentrations ranged from those typical in ‘pristine’ environments to those found in ‘impacted’ sites (e.g., Graham et al. 2011; Pruden et al. 2006).

**Table 2:** Gene content (*log-*transformed) in samples collected throughout Western Australia

	Absolute abundance *log*(genes/g soil)		Relative abundance *log*(genes/16S rRNA)	
	Mean (95% CI)	Min/max	Mean (95% CI)	Min/max
16S rRNA	9.32 (0.17)	7.11/10.06		
*bla*_TEM_	6.41 (0.22)	2.60/8.42	−3.01 (0.22)	−7.31/−0.35
*bla*_CTX_	3.46 (0.11)	1.65/4.00	−4.04 (0.16)	−8.07/−3.67
*bla*_OXA_	5.00 (0.09)	3.50/6.38	−4.33 (0.17)	−6.38/−1.79
*bla*_SHV_	5.11 (0.23)	2.92/6.47	−4.16 (0.23)	−8.03/−2.12
Tet1	4.67 (0.29)	2.36/6.32	−4.40 (0.30)	−7.28/−1.29
Tet2	5.59 (0.37)	2.65/7.98	−3.47 (0.38)	−7.03/−1.80
Tet3	5.51 (0.24)	3.05/7.37	−3.65 (0.25)	−6.95/−1.85
Tet4	6.63 (0.14)	4.26/7.72	−2.69 (0.18)	−5.47/−1.51
*tet*(M)	5.89 (0.14)	4.20/7.27	−3.47 (0.20)	−5.70/−1.01
*tet*(W)	4.48 (0.20)	1.89/7.19	−5.15 (0.25)	−7.75/−2.44
*sul*1	5.02 (0.30)	1.25/6.72	−4.48 (0.31)	−8.37/−1.74
*sul*2	4.85 (0.19)	1.29/6.59	−4.58 (0.21)	−8.48/−2.04
*sul*3	4.79 (0.19)	2.66/7.24	−4.51 (0.24)	−7.04/−1.71

### Bivariate correlations—absolute abundances

We assessed the relationships between metals and the abundances of AR genes in the soils to determine whether metals were likely to influence AR gene presence. Examining absolute gene abundances, representing the amount of genes per gram of soil, correlational analyses (Table 3) found a number of genes related to metal concentrations. In particular, manganese and vanadium levels exhibited the greatest number of significant correlations to AR genes: *bla*_CTX_, *bla*_OXA_, *bla*_TEM_, tet4 series, *tet*M, *tet*W, *sul*1 and *sul*2; however, they simultaneously correlated with 16S rRNA genes (Mn, *r* = 0.261; *p* = 0.013; V, *r* = 0.211, *p* = 0.046), suggesting co-linearity of data. Correlation values may reflect increasing numbers of bacteria overall, with similar proportions of the communities having the genes. Ignoring the metals that had correlations with 16S rRNA genes (which also included nickel *r* = 0.199, *p* = 0.061), significant correlations were found with copper (with *bla*_OXA_, *bla*_TEM_, *tet*M) and aluminium (*bla*_OXA_, *bla*_TEM_, *tet*M, *tet*W, *sul*2 and *sul*3).

**Table 3:** Significant bivariate correlations between metal content and relative AR gene abundances (normalised to 16S rRNA gene abundance)

	Absolute (unnormalised) gene relationships		Relative (normalised to 16S) gene relationships	
*bla*_TEM_	Al Cu Mn Ni U V Zn	(*r* = 0.29; *p* = 0.01) (*r* = 0.26; *p* = 0.02) (*r* = 0.45; *p* < 0.01) (*r* = 0.24; *p* = 0.03) (*r* = 0.19; *p* = 0.08) (*r* = 0.32; *p* < 0.01) (*r* = 0.30; *p* = 0.01)	Al Mn Pb U	(*r* = 0.20; *p* = 0.08) (*r* = 0.20; *p* = 0.07) (*r* = 0.22; *p* = 0.05) (*r* = 0.22; *p* = 0.05)
*bla*_CTX_	Mn V U	(*r* = 0.29; *p* = 0.01) (*r* = 0.23; *p* = 0.05) (*r* = 0.23; *p* = 0.05)	U	(*r* = 0.30; *p* = 0.01)
*bla*_OXA_	Al Cu Mn Ni Pb V Zn	(*r* = 0.32; *p* < 0.01) (*r* = 0.31; *p* < 0.01) (*r* = 0.38; *p* < 0.01) (*r* = 0.18; *p* = 0.09) (*r* = 0.25; *p* = 0.02) (*r* = 0.27; *p* = 0.01) (*r* = 0.35; *p* < 0.01)	Pb	(*r* = 0.26; *p* = 0.01)
*bla*_SHV_	As	(*r* = 0.20; *p* = 0.07)	As Ni V	(*r* = 0.28; *p* = 0.01) (*r* = −0.28; *p* = 0.01) (*r* = −0.20; *p* = 0.06)
Tet1				
Tet2	Hg Pb V	(*r* = 0.25; *p* = 0.02) (*r* = −0.25; *p* = 0.02) (*r* = −0.18; *p* = 0.10)	Hg Mn Ni Pb Se V	(*r* = 0.23; *p* = 0.03) (*r* = −0.21; *p* = 0.06) (*r* = −0.25; *p* = 0.02) (*r* = −0.18; *p* = 0.10) (*r* = −0.19; *p* = 0.09) (*r* = −0.27; *p* = 0.01)
Tet3			Se	(*r* = −0.19; *p* = 0.07)
Tet4	Mn V	(*r* = 0.23; *p* = 0.03) (*r* = 0.21; *p* = 0.05)		
*tet*(M)	Al Mn Cu Zn	(*r* = 0.20; *p* = 0.09) (*r* = 0.26; *p* = 0.03) (*r* = 0.24; *p* = 0.04) (*r* = 0.26; *p* = 0.03)	Co	(*r* = 0.19; *p* = 0.09)
*tet*(W)	Al Mn V Zn	(*r* = 0.27; *p* = 0.02) (*r =* 0.40; *p* < 0.01) (*r =* 0.32; *p* = 0.01) (*r* = 0.26; *p* = 0.03)		
*sul*1	Mn V	(*r* = 0.27; *p* = 0.01) (*r* = 0.18; *p* = 0.09)		
*sul*2	Al Mn V	(*r* = 0.19; *p* = 0.09) (*r* = 0.30; *p* = 0.01) (*r* = 0.33; *p* < 0.01)		
*sul*3	Al	(*r* = 0.19; *p* = 0.09)		

Both variables were log-transformed to distribute the data better prior to correlation analysis

Genes *bla*_OXA_ and *bla*_TEM_ had the greatest number of positive relationships with metal concentrations: aluminium, copper, manganese, nickel, vanadium and zinc; *bla*_TEM_ also correlated with uranium concentrations, whereas *bla*_OXA_ correlated with lead. Additional correlations included *bla*_SHV_ and arsenic, tet2 series and mercury and *bla*_CTX_ and uranium. There were some negative correlations among tet2 notably with lead (*r* = −0.249, *p* = 0.020) and vanadium (*r* = −0.177, *p* = 0.100), which suggested that specific populations could have been negatively impacted by these metals.

Copper and zinc have been reported in literature to be strong contributors to absolute abundance of resistance traits in soils, having direct correlations with beta-lactam (Hölzel et al. 2012; Hu et al. 2016; Knapp et al. 2011), sulphonamide (Hu et al. 2016; Ji et al. 2012), erythromycin (Knapp et al. 2011) and tetracycline resistance (Knapp et al. 2011; Peltier et al. 2010). Bacterial cells biochemically require these elements, although elevated levels are toxic; as such, they have mechanisms to maintain homeostasis in cells, and some traits that promote tolerance to higher concentrations can be found on mobile genetic elements along with antibiotic resistance genes (e.g., Hasman and Aarestrup 2005). While this study supports previous studies with positive correlations found between copper and/or zinc and the number of antibiotic resistance genes, most studies examined elevated levels of metal pollution. Here, environmental conditions with lower levels of metals were examined.

### Bivariate correlations—relative (per total bacteria) abundances

When normalised to 16S rRNA, the correlation patterns changed, and many relationships became less apparent, which may not be surprising given the prevalence of ‘lower’ metal concentrations. However, *bla*_TEM_/16S continued to be impacted by metal concentrations with positive correlations with aluminium, manganese, lead and uranium. Correlations remained significant among metals and other beta-lactam resistance genes: arsenic and *bla*_SHV_/16S, lead and *bla*_OXA_/16S and uranium and *bla*_CTX_/16S.

Limited correlations between relative gene abundances and soil character were found. Most correlations were weak and insignificant, except negative correlations were found with *bla*_OXA_/16S (*r* = −0.338, *p* = 0.010), *tet*M/16S (*r* = −0.406, *p* = 0.005), *sul*2/16S (*r* = 0.296, *p* = 0.028) and *sul*3/16S (*r* = −0.289, *p* = 0.036).

The decrease in number of correlated pairs may be attributed to the relatively low metal concentrations in this study (as compared to many found in literature); the remaining pairwise relationships were likely to have been toxic. With the exception of manganese, these metals had no (or very limited) nutritional benefit to bacteria. Bacterial responses to metals, like antibiotics, are concentration dependent (Bernier and Surette 2013). At lower levels, some elements provide micronutrition for biochemical reactions as essential enzyme co-factors. At elevated concentrations, they become toxic, and exposure can lead to series of possible cellular stress responses (gene expression), community adaptation (including mutation and resistance development) or cell death.

There would be fewer strategies for cells to detoxify non-essential elements, which can adversely impact microbial populations. In this case, survivors would have developed or acquired specific resistance traits. The bias in correlation patterns appeared to be related to whether the metals contribute a nutritional need at lower concentrations. As mentioned previously, manganese, copper and zinc contribute to absolute gene abundances, suggesting that the cells are better adapted to survive at higher concentrations by biochemical mechanisms that exist (either innate or acquired) to assist the sequestration and handling of metals. In contrast, there are fewer adaptations for the non-nutritional metals, and populations would consequently become stressed.

Chromium, lead, arsenic, mercury, nickel and iron have been previously found related to ARG (Ji et al. 2012; Knapp et al. 2011; Timoney et al. 1978). However, there has been inconsistency in results with both positive and negative correlations being reported. For example, Hölzel et al. (2012) found that mercury and lead, which are not associated with known biochemical reactions, inhibited bacterial population growth rather than resistance development. In this study, aluminium, vanadium and lead (and to a limited extent, uranium) impacted microbial communities.

### Multiple linear regression models

**Fig. 1:**
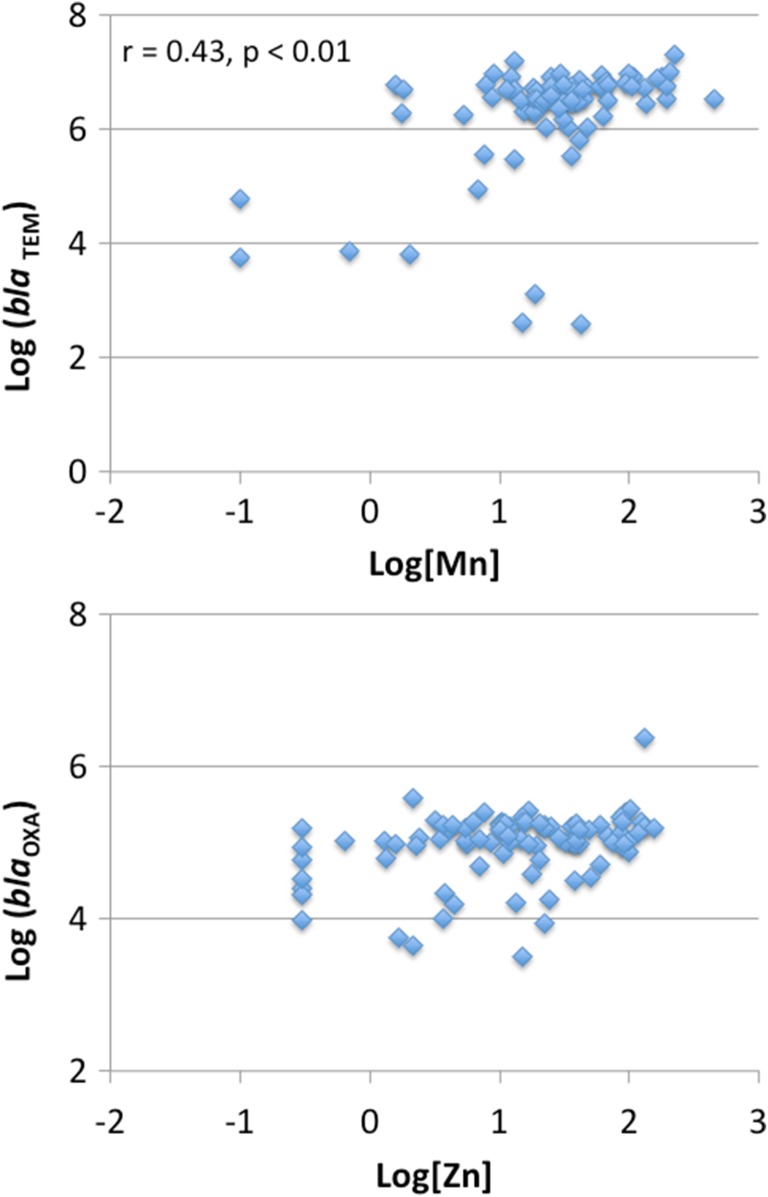
Exemplar scatterplots demonstrating typical heteroscedastic patterns in bivariate analysis

While significant relationships existed between AR and potential toxic metals in bivariate analysis, correlation coefficients (*r* values) were low. The complexity of soil environments makes it difficult to parameterise all possible factors that might contribute to AR (Knapp et al. 2011). Upon examination of scatterplots (see Fig. 1), higher range of gene values (normalised to 16S rRNA) existed at lower concentrations; for example, elevated proportions of bacteria having ARG may be present despite potentially low exposure to a particular metal, suggesting that another element or factor may be impacting the populations. With bivariate correlations, exposure-related effects may not become apparent until the contribution to AR by an individual metal exceeds the contribution by other factors. As such, the heteroscedastic nature of the data suggests that multiple factors influence AR gene abundances. For this reason, we also examined multiple linear regression (MLR) patterns.

**Fig. 2:**
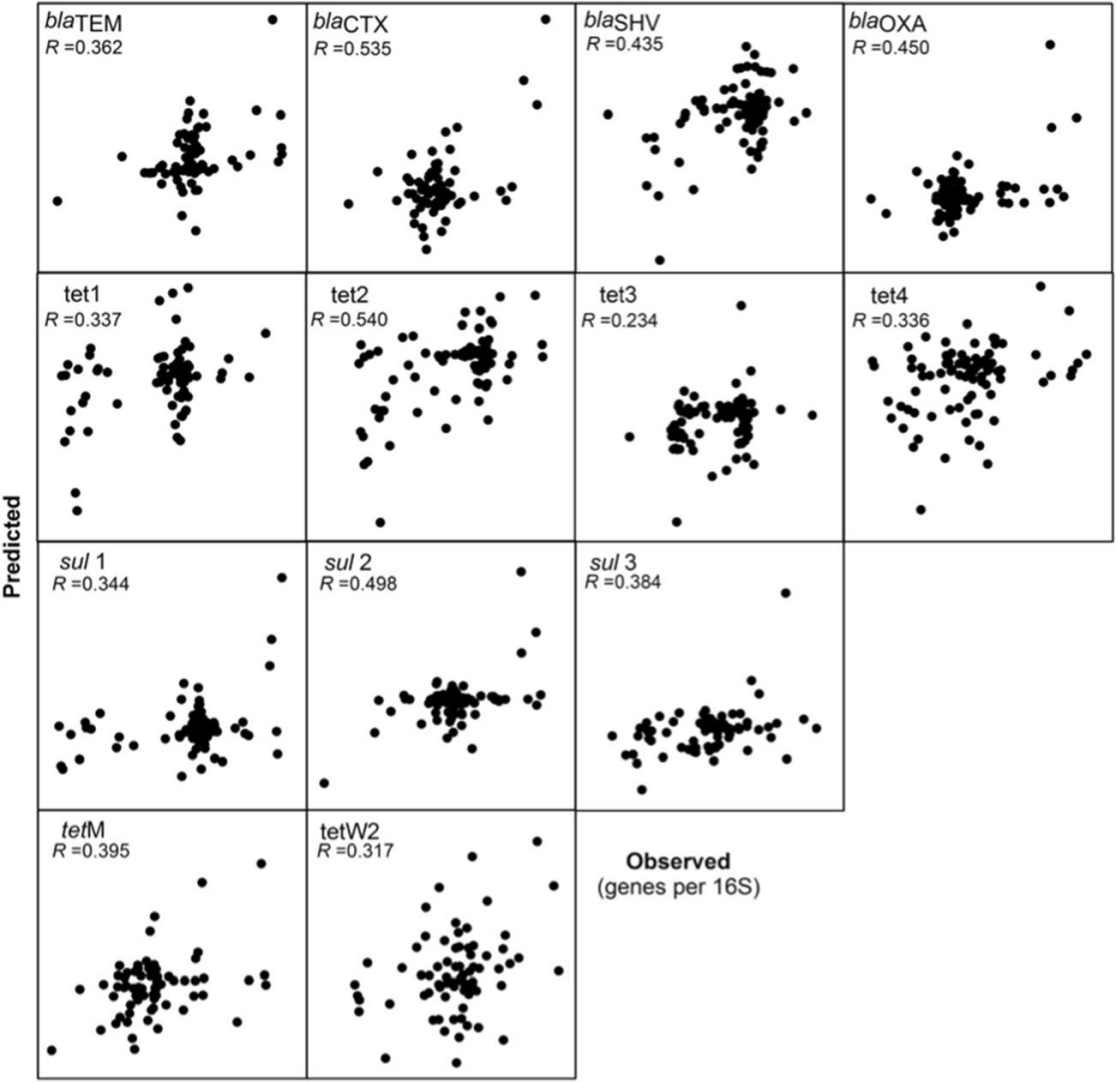
Observed versus predicted values (per 16S rRNA values) based on multilinear regression analysis. Significant (*p* < 0.01) patterns presented

The following seven metals were entered into a MLR model: aluminium, copper, manganese, nickel, lead and zinc based on their bivariate relationships. We used the model to predict relative gene abundances, as we focused on gene selection. More strongly significant correlations were found (Table 4; Fig. 2): *bla*_CTX_ (*R* = 0.54, *p* < 0.01), *bla*_OXA_ (*R* = 0.45, *p* = 0.01), *bla*_SHV_ (*R* = 0.44, *p* = 0.02), tet2 gene series (*R* = 0.54, *p* < 0.01), *sul*2 (*R* = 0.50, *p* < 0.01) and *sul*3 (*R* = 0.38, *p* = 0.10).

**Table 4:** Multiple linear regression models for gene predictions

Dependent variable	Correlation (*R*)	*F* score	Significance (*p* value)	Model:	Coefficients:						
				Constant	Al	Cu	Mn	Ni	Pb	Se	Zn
*bla*_TEM_/16S	0.362	1.57	0.157	−3.202	0.00001	0.0133	**0.00388**	−0.0317	0.0242*	**−0.00619**	−0.00531
*bla*_CTX_/16S	0.535	3.715	0.002	−6.230	0.00015*	−0.0123	0.00120	−0.0409	**0.0169**	0.00545*	−0.0047
*bla*_OXA_/16S	0.450	2.898	0.009	−4.437	0.00011*	−0.00059	−0.00108	−0.0506	0.0182*	0.00191	−0.00387
*bla*_SHV_/16S	0.435	2.696	0.015	−4.068	**0.00011**	0.0154	**−0.00425**	−0.111*	−0.00335	0.00704*	−0.00014
*Tet1*	0.337	1.132	0.254	−4.222	0.00010	−0.0464	−0.00322	**−0.0975**	0.00175	0.00383	0.00993
*Tet2*	0.540	4.645	<0.001	−3.093	**0.00016**	**0.0802**	−0.0106*	−0.0385	−0.0209	0.00309	−0.0146*
*Tet3*	0.234	0.654	0.710	−3.481	−0.00001	−0.0259	−0.00173	−0.0199	0.0138	−0.00019	0.00122
*Tet4*	0.336	1.456	0.195	−2.622	0.00001	−0.0110	−0.00195	−0.0534	0.0128	0.00214	−0.00086
*tetM*	0.421	1.965	0.074	−3.551	0.00014*	0.0113	−0.00138	**−0.0976**	0.00678	0.00241	−0.00294
*tetW*	0.317	1.056	0.402	−5.231	0.00013	0.0202	0.00348	−0.109	0.00753	−0.00351	−0.00705
*sul1*	0.344	1.453	0.197	−4.602	0.00022*	−0.0280	0.00168	−0.0331	−0.00547	−0.00076	−0.00064
*sul2*	0.498	3.670	0.002	−4.564	0.00013*	−0.0402*	0.00034	−0.0251	0.0206*	0.00066	−0.00349
*sul3*	0.384	1.780	0.105	−5.511	**0.00010**	−0.0167	−0.00305	−0.0142	**0.0199**	0.00295	−0.00318

Significant predictors, their coefficients and *p* value are included; coefficients in bold represent significant (*p* < 0.05) contributions. The *R* represents the coefficient of determination for the entire model; parameters entered into the model included aluminium, copper, manganese, nickel, lead, selenium and zinc

When combining factors in a multilinear regression model, the relationship between soil heavy metal conditions and antibiotic resistance improved, suggesting that multiple stressors may additively drive the selection of resistance. Here, even at the low metal concentrations in the residential soils in this study, approximately 25% of the variation in the incidence of genetic markers of antibiotic resistance was explained by the models (Table 4).

### Link between metals and antibiotic resistance

The linkage between antibiotic resistance and metal exposure has been known for many decades, when it was first discovered that penicillinase was linked with mercury exposure (Fraser 1971; Richmond et al. 1964). Two mechanisms are involved in the possible linkages. Cross-resistance involves a single gene that confers resistance to both the antibiotic and metal, e.g., an efflux pump. Co-resistance is when separate traits are closely linked, e.g., on a transferable genetic element or operon, and are transferred together (e.g., Richmond et al. 1964). In both cases, metal exposure is sufficient to select and maintain AR genotype (Baker-Austin et al. 2006).

Some metals can directly trigger the resistance phenotype. Tetracycline resistance genes *tet*B, *tet*C and *tet*D (tet1 series) and *tet*A, *tet*E and *tet*G (tet2 series) represent tetracycline-metal porters (efflux pumps) and are known to affect divalent cations in cells [e.g., Zn^+2^ and Cu^+2^; (Yamaguchi et al. 1990)], and the metal-tetracycline complex is capable of mediating the activity of tetR, a repressor protein associated with tet determinants in gram-negative bacteria (Palm et al. 2008). Our results confirm the linkage between tetracycline-resistant genes and the divalent cations Zn^+2^ and Cu^+2^, as well as Mn^+2^.

On the other hand, *tet*M and *tet*W, along with *tet*O, *tet*Q and *tet*S (part of the tet3 and tet4 series), are ribosomal protection proteins. While the exact biochemical mechanism(s) for co-resistance are not known, they have been reported to be associated with metal pollution (Berg et al. 2005; Knapp et al. 2011). Many of these genes, however, are associated with conjugative transposons (e.g., *Tn*917 and the *Tn*916–1525 family), which are often associated with multiple resistance (Roberts 2012). Beta-lactam resistance has been associated with mercury (e.g., Fraser 1971; Richmond et al. 1964) and copper (e.g., Knapp et al. 2011) exposure, and lead exposure contributed to AR in enteric bacteria in poultry (Nisanian et al. 2014). The results from our study indicate linkage with other metals, namely aluminium, manganese, uranium and lead. Correlations have been reported between *sul* genes and copper, zinc and mercury in wastewater (Ji et al. 2012), whereas we again did not find a relationship with mercury, although there was a correlation with manganese in addition to aluminium and vanadium. Soil mercury concentrations in this study were very low (with over 80% of samples <LOD), and this is likely to have precluded detection of a relationship between this metal and AR genes.

There is no information on previous management of the garden soils, which is unfortunate. Some garden amendments could contain both metals and ARG. However, the results of this study suggest that there are relationships, although weak, between metals and ARG concentration. Improvements to the study, and certainly worth considering for future experiments, include sequential metal extractions and some indication of their bioavailability and also the determination of threshold toxicity, especially among metals that have nutritional importance, and determination of whether resistance traits are innate to the cell or acquired, which may carry a greater risk of transfer. Here, given the nature of the archived soils, it would have been impossible to conduct these analyses. However, the results do suggest an avenue further research, especially since landscape contributors to ARG and basal levels in the environment remain poorly understood.

## Conclusions

It is known that the presence of metals, at sufficient concentrations, results in metal tolerance (or resistance), which has (in turn) been proven to link to antibiotic resistance. This suggests that AR traits will be selected by metals, even in the absence of antibiotics. This study demonstrated that even at low concentrations of metals (including aluminium, copper, manganese and lead) in residential soils, antibiotic resistance was selected, as evidenced by increased relative gene abundances.

Many aspects of antimicrobial resistance have to be considered when studying traits in the environment, and one must consider the multitude of factors, including possible synergistic effects. Evidence here suggests that metal conditions and the presence of potentially toxic metals make a contribution to the spread of antimicrobial resistance. There are two possible cases for increased resistance development: (1) The amount of genes can increase, along with the amount of total bacteria, as evident following exposure to biochemical relevant elements, (2) or the increased selection of bacteria with resistance traits. The measurement type, whether absolute gene counts or relative abundances, can possibly explain elevated resistance.

## Abbreviations

16S rRNA: 16S ribosomal ribonucleic acid
AR: Antibiotic resistance
ARG: Antibiotic resistance genes
*bla*: Beta-lactamase gene (-TEM, -SHV, -OXA and -CTX each represent a type of gene)
ICP-OES: Inductively coupled plasma optical emission spectrometry
PCR: Polymerase chain reaction
qPCR: Quantitative polymerase chain reaction
*sul*: Sulphonamide resistance gene
*tet*: Tetracycline resistance gene

## References

[CR1] Abella J, Fahy A, Duran R, Cagnon C (2015) Integron diversity in bacterial communities of freshwater sediments at different contamination levels. FEMS Microbiol Ecol 91: fiv140 10.1093/femsec/fiv140 26552833

[CR2] Alonso A, Sanchez P, Martinez JL (2001). Environmental selection of antibiotic resistance genes. Environ Microbiol.

[CR3] Ashbolt NJ, Amezquita A, Backhaus T, Borriello P, Brandt KK, Collignon P, Coors A, Finley R, Gaze WH, Heberer T, Lawrence JR, Larsson DGJ, McEwan SA, Ryan JJ, Schönfeld J, Silley P, Snape JR, Van den Eede C, Topp E (2013). Human health risk assessment (HHRA) for environmental development and transfer of antibiotic resistance. Environ Health Perspect.

[CR4] Australian Institute of Health and Welfare (2004) Rural, regional and remote health: a guide to remoteness classifications http://www.aihw.gov.au/WorkArea/DownloadAsset.aspx?id=6442459567. Last accessed 04 August 2016.

[CR5] Baker-Austin C, Wright MS, Stepanauskas R, McArthur JV (2006). Co-selection of antibiotic and metal resistance. Trends Microbiol.

[CR6] Bass L, Liebert CA, Lee MD, Summers AO, White DG, Thayer SG, Maurer JJ (1999). Incidence and characterization of integrons, genetic elements mediating multiple-drug resistance, in avian *Escherichia coli*. Antimicrob Agents Chemother.

[CR7] Beaber JW, Hochhut B, Waldor MK (2004). SOS response promotes horizontal dissemination of antibiotic resistance genes. Nature.

[CR8] Berendock TU, Manaia CM, Merlin C, Fatta-Kassinos D, Cytryn E, Walsh F, Bürgmann H, Sørum H, Norström M, Pons M-N, Kruenzinger N, Huovinen P, Stefani S, Schwartz T, Kisand V, Baquero F, Martinez JL (2015). Tackling antibiotic resistance: the environmental framework. Nat Rev Microbiol.

[CR9] Berg J, Thorsen MK, Holm PE, Jensen J, Nybroe O, Brandt KK (2010). Cu exposure under field conditions coselects for antibiotic resistance as determined by a novel cultivation-independent bacterial community tolerance assay. Environ Sci Technol.

[CR10] Berg J, Tom-Petersen A, Nybroe O (2005). Copper amendment of agricultural soil selects for bacterial antibiotic resistance in the field. Lett Appl Microbiol.

[CR11] Bernier SP, Surette MG (2013) Concentration-dependent activity of antibiotics in natural environments. Front Microbiol 4 10.3389/fmicb.2013.00020 PMC3574975 23422936

[CR12] Callan AC, Hinwood AL, Ramalingam M, Boyce M, Heyworth J, McCafferty P, Odland JO (2013). Maternal exposure to metals-concentrations and predictors of exposure. Environ Res.

[CR13] Canton R (2009). Antibiotic resistance genes from the environment: a perspective through newly identified antibiotic resistance mechanisms in the clinical setting. Clin Microbiol Infect.

[CR14] Davies J, Davies D (2010). Origins and evolution of antibiotic resistance. Microbiol Mol Biol Rev.

[CR15] Day PR, Black CA (1965). Particle fractionation and particle-size analysis. Methods of soil analysis, part 1.

[CR16] Dhakephalkar PK, Chopade BA (1994). High-levels of multiple metal resistance and its correlation to antibiotic-resistance in environmental isolates of Acinetobacter. Biometals.

[CR17] Dorn-In S, Bassitta R, Schwaiger K, Bauer J, Hölzel CS (2015). Specific amplification of bacterial DNA by optimized so-called universal bacterial primers in samples rich of plant DNA. J Microbiol Methods.

[CR18] Fraser WR (1971). Some features of antibiotic resistance in Staphylococci: mercury resistance and multiple antibiotic resistance. Proc Roy Soc Med.

[CR19] Ghosh A, Singh A, Ramteke PW, Singh VP (2000). Characterization of large plasmids encoding resistance to toxic heavy metals in Salmonella abortus equi. Biochem Biophys Res Commun.

[CR20] Graham DW, Olivares-Rieumont S, Knapp CW, Lima L, Werner D, Bowen E (2011). Antibiotic resistance gene abundances associated with waste discharges to the Almendares River near Havana, Cuba. Environ Sci Technol.

[CR21] Guo XC, Liu S, Wang Z, Zhang XX, Li M, Wu B (2014). Metagenomic profiles and antibiotic resistance genes in gut microbiota of mice exposed to arsenic and iron. Chemosphere.

[CR22] Hasman H, Aarestrup FM (2005). Relationship between copper, glycopeptide, and macrolide resistance among Enterococcus faecium strains isolated from pigs in Denmark between 1997 and 2003. Antimicrob Agents Chemother.

[CR23] Hinwood AL, Callan AC, Ramalingam M, Boyce M, Heyworth J, McCafferty P, Odland JO (2013). Cadmium, lead and mercury exposure in non-smoking pregnant women. Environ Res.

[CR24] Hölzel CS, Muller C, Harms KS, Mikolajewski S, Schafer S, Schwaiger K, Bauer J (2012). Heavy metals in liquid pig manure in light of bacterial antimicrobial resistance. Environ Res.

[CR25] Hu HW, Wang JT, Li J, Li JJ, Ma YB, Chen D, He JZ (2016). Field-based evidence for copper contamination induced changes of antibiotic resistance in agicultural soils. Environ Microbiol.

[CR26] Ji XL, Shen QH, Liu F, Ma J, Xu G, Wang YL, Wu MH (2012). Antibiotic resistance gene abundances associated with antibiotics and heavy metals in animal manures and agricultural soils adjacent to feedlots in Shanghai, China. J Hazard Mater.

[CR27] Knapp CW, Dolfing J, Ehlert PAI, Graham DW (2010). Evidence of increasing antibiotic resistance gene abundances in archived soils since 1940. Environ Sci Technol.

[CR28] Knapp CW, Lima L, Olivares-Rieumont S, Bowen E, Werner D, Graham DW (2012) Seasonal variations in antibiotic resistance gene transport in the Almendares River, Havana. Cuba Front Microbiol 3 10.3389/fmicb.2012.00396 PMC3505016 23189074

[CR29] Knapp CW, McCluskey SM, Singh BK, Campbell CD, Hudson G, Graham DW (2011) Antibiotic resistance gene abundances correlate with metal and geochemical conditions in archived scottish soils PLoS One 6 10.1371/journal.pone.0027300 PMC3212566 22096547

[CR30] Li J, Ma YB, Hu HW, Wang JT, Liu YR, He JZ (2015). Field-based evidence for consistent responses of bacterial communities to copper contamination in two contrasting agricultural soils. Front Microbiol.

[CR31] Marques AM, Congregado F, Simonpujol DM (1979). Antibiotic and heavy-metal resistance of Pseudomonas aeruginosa isolated from soils. J Appl Bacteriol.

[CR32] Martinez JL (2008). Antibiotics and antibiotic resistance genes in natural environments. Science.

[CR33] Martinez JL (2009). Environmental pollution by antibiotics and by antibiotic resistance determinants. Environ Pollut.

[CR34] McLaughlin MJ, Lofts S, Warne M, Amorim MJB, Fairbrother A, Lanno R, Hendershot W, Schelkat CE, Ma Y, Paton GJ, Merrington G, Schoeters I (2010). Derivation of ecological based soil standards for trace elements. Soil quality standards.

[CR35] NEPC (1999) National Environment Protection (Assessment of Contaminated Land) Measure 1999. Schedule B (7a) Guideline on Health Investigation Levels. National Environment Protection Council.

[CR36] Ng LK, Martin I, Alfa M, Mulvey M (2001). Multiplex PCR for the detection of tetracycline resistant genes. Mol Cell Probes.

[CR37] Nisanian M, Holladay SD, Karpuzoglu E, Kerr RP, Williams SM, Stabler L, Vaun McArthur J, Tuckfield RC, Gogal RM (2014). Exposure of juvenile Leghorn chickens to lead acetate enhances antibiotic resistance in enteric bacterial flora. Poult Sci.

[CR38] Palm GJ, Lederer T, Orth P, Saenger W, Takahashi M, Hillen W, Hinrichs W (2008). Specific binding of divalent metal ions to tetracycline and to the tet repressor/tetracycline complex. J Biol Inorg Chem.

[CR39] Peak N, Knapp CW, Yang RK, Hanfelt MM, Smith MS, Aga DS, Graham DW (2007). Abundance of six tetracycline resistance genes in wastewater lagoons at cattle feedlots with different antibiotic use strategies. Environ Microbiol.

[CR40] Pei RT, Kim SC, Carlson KH, Pruden A (2006). Effect of river landscape on the sediment concentrations of antibiotics and corresponding antibiotic resistance genes (ARG). Water Res.

[CR41] Peltier E, Vincent J, Finn C, Graham DW (2010). Zinc-induced antibiotic resistance in activated sludge bioreactors. Water Res.

[CR42] Perry J, Wright G (2013). The antibiotic resistance "mobilome": searching for the link between environment and clinic. Front Microbiol.

[CR43] Pruden A, Pei RT, Storteboom H, Carlson KH (2006). Antibiotic resistance genes as emerging contaminants: studies in northern Colorado. Environ Sci Technol.

[CR44] Richmond MH, Parker M, Jevons MP, John M (1964) High penicillinase production correlated with mulitiple antibiotic resistance in *Staphylococcus aureus*. Lancet: 293–296 10.1016/s0140-6736(64)92407-9 14089055

[CR45] Rhoades JD (1982) Soluble salts, methods of soil analysis, part 2. Chemical and Microbiological Properties. American Society of Agronomy Monograph No. 9, 2nd ed.

[CR46] Roberts M (2012) Acquired tetracycline resistance genes. In: Dougherty T, Pucci M (Eds.), Antibiotic Discovery and Development, pp 543–568

[CR47] Schollenberger CJ (1945). Determination of soil organic matter. Soil Sci.

[CR48] Schulte EE, Hopkins BG (1996) Estimation of soil organic matter by weight loss-on-ignition. pp 21–31

[CR49] Seiler C, Berendonk TU (2012) Heavy metal driven co-selection of antibiotic resistance in soil and water bodies impacted by agriculture and aquaculture Front Microbiol 3 10.3389/fmicb.2012.00399 PMC3522115 23248620

[CR50] Shlaes DM (2010). Antibiotics.

[CR51] Sheldrick BH, Wang C (1993). Soil sampling and methods of analysis.

[CR52] Smith MS, Yang RK, Knapp CW, Niu YF, Peak N, Hanfelt MM, Galland JC, Graham DW (2004). Quantification of tetracycline resistance genes in feedlot lagoons by real-time PCR. Appl Environ Microbiol.

[CR53] Stepanauskas R, Glenn TC, Jagoe CH, Tuckfield RC, Lindell AH, King CJ, McArthur JV (2006). Coselection for microbial resistance to metals and antibiotics in freshwater microcosms. Environ Microbiol.

[CR54] Stepanauskas R, Glenn TC, Jagoe CH, Tuckfield RC, Lindell AH, McArthur JV (2005). Elevated microbial tolerance to metals and antibiotics in metal-contaminated industrial environments. Environ Sci Technol.

[CR55] Su JQ, Wei B, Ou-Yang WY, Huang FY, Zhao Y, Xu HJ, Zhu Y-G (2015). Antibiotic resistome and its association with bacterial communities during sewage sludge composting. Environ Sci Technol.

[CR56] Tenover FC (2006). Mechanisms of antimicrobial resistance in bacteria. Amer J Infect Cont.

[CR57] Timoney JF, Port J, Giles J, Spanier J (1978). Heavy-metal and antibiotic resistance in bacterial flora of sediments of New York bight. Appl Environ Microbiol.

[CR58] USEPA (2002) Method 3050B. Acid Digestion of Sediment, Sludges and Soils. US Environmental Protection Agency, Washington DC

[CR59] Van Reeuwijk LP (2002). Procedures for soil analysis (6th edition).

[CR60] WHO (2014) Global Report on Surveillance. World Health Organisation.

[CR61] Wright MS, Peltier GL, Stepanauskas R, McArthur JV (2006). Bacterial tolerances to metals and antibiotics in metal-contaminated and reference streams. FEMS Microbiol Ecol.

[CR62] Yamaguchi A, Udagawa T, Sawai T (1990). Transport of divalent cations with tetracycline as mediated by the transposon Tn10-encoded tetracycline resistance protein. J Biol Chem.

[CR63] Zhu Y-G, Johnson TA, Su JQ, Qiao M, Guo GX, Stedfeld RD, Hashsham SA, Tiedje JM (2013). Diverse and abundant antibiotic resistance genes in Chinese swine farms. Proc Natl Acad Sci U S A.

